# Client Satisfaction with Delivery Care Service and Associated Factors in the Public Health Facilities of Gamo Gofa Zone, Southwest Ethiopia:* In a Resource Limited Setting*

**DOI:** 10.1155/2016/5798068

**Published:** 2016-06-20

**Authors:** Rahel Tesfaye, Amare Worku, Wanzahun Godana, Bernt Lindtjorn

**Affiliations:** ^1^Arba Minch College of Health Sciences, P.O. Box 155, Arba Minch, Ethiopia; ^2^Addis Continental Institute of Public Health, P.O. Box 1140, Addis Ababa, Ethiopia; ^3^Department of Public Health, Arba Minch University, P.O. Box 21, Arba Minch, Ethiopia; ^4^Centres for International Health, University of Bergen, Bergen, Norway

## Abstract

*Background*. Ensuring patient satisfaction is an important means of secondary prevention of maternal mortality. This study presents findings from a multidimensional study of client satisfaction from the Gamo Gofa Zone in Southwest Ethiopia.* Methods*. A facility based cross-sectional study using exit interviews was conducted from 2014. Client satisfaction was measured using a survey adopted from the Donabedian quality assessment framework. Thirteen health institutions were randomly sampled of 66 institutions in Gamo Gofa Zone. Logistic regression was used to determine predictors of client satisfaction.* Results*. The overall satisfaction level of the clients in this study was 79.1% with (95% CI; 75–82). Women attending health centres were more likely satisfied than women attending hospitals (*χ* ^2^ = 83.7, *df* = 12, *P* < 0.001). The proportion of women who complained about an unfriendly attitude or unresentful care from health workers was higher in the hospitals (*χ* ^2^ = 27.4, *df* = 1, *P* < 0.001). The presence of support persons during child birth improved client satisfaction (AOR = 6.23 95% CI; 2.75–14.1) and women who delivered with caesarean section are four times more likely satisfied than those who deliver vaginally (AOR 3.6 95% CI; 1.44–9.06). Client satisfaction was reduced if the women had to pay for the services (AOR = 0.27 95% CI; 0.09–81).* Conclusions*. The study shows that overall satisfaction level is good. More emphasis should be put on giving women friendly care, particularly at the hospitals.

## 1. Background

Client satisfaction among pregnant women attending health delivery institutions is an important measure to assess quality of health care [1, 2]. Providing quality service means meeting client expectations, which is a function of their experiences during a given service encounter [3]. The World Health Organization (WHO) emphasizes ensuring patient satisfaction as a means of secondary prevention of maternal mortality since satisfied women may be more likely to adhere to health providers' recommendations [4].

Over the last decade, there has been an increasing effort to address the discrepancies between the delivery of health care and client needs [5]. Despite the international emphasis to address the unmet health needs of pregnant women and children progress in reducing maternal mortality has been slow. This is particularly worrying Sub-Saharan Africa where most maternal deaths occur every year because of absence of quality healthcare [6, 7]. In Ethiopia, mortality ratio (MMR) is 676 per 1000 live births, and the percentage of births delivered at public health institutions is less than 10 [8]. To improve maternal health services, we need to improve health care to communities, with increased volume, speed, and quality [9].

Different studies from health delivery institutions show that client care satisfaction levels range from 69% to 83% [10, 11]. Little is known about client satisfaction at public health facilities in the Gamo Gofa Zone in southwest Ethiopia. Therefore, the objective of this study is to determine the level of client satisfaction at health delivery institutions. We believe such a study would provide valuable information to health care providers, local administrators, and policy makers to improve services in Ethiopia.

## 2. Materials and Methods

### 2.1. Study Design and Setting

A facility based cross-sectional study was conducted from December 17, 2013, to January 27, 2014, in thirteen public health facilities of Gamo Gofa Zone. The capital town of the zone, Arba Minch, is located about 500 km southwest of Addis Ababa. In 2013 the population of the zone was 1.847.264 people; of them 440, 449 were women in reproductive age. The zone has two town administrations and 15 woredas (districts) with three hospitals and 63 health centers which provide delivery services.

### 2.2. Sample Size Determination

Using the results from previous studies with client satisfaction of 83%, the sample size was calculated using Epi Info Version 7 with the following assumptions: 80% power, 95% confidence level, odd ratio 2, design effect of 1.5, and 5% nonresponse rate. The estimated sample size was thus 435 women.

### 2.3. Sampling Technique

We randomly selected 13 health institutions for the study from a sampling frame of 66 health institutions in Gamo Gofa Zone. The total number of delivering mothers in each health institution was calculated based on proportional to the number of deliveries attended at the health institutions in the last three months. Finally data was collected from every other woman who delivered in those health facilities till the required sample size was reached.

### 2.4. Data Collection Procedures

Data was collected using a survey instrument adopted from the Donabedian quality assessment framework [12]. Thirteen unemployed midwives were initially trained and later collected the data, and 3 persons with B.S. degrees in public health supervised the data collection. The interviews were done when the patient was discharged from the institution. It was conducted in Amharic language after the questions were pretested and necessary modifications were made.

### 2.5. Data Analysis

The Statistical Package for Social Sciences version 16.0 (SPSS Inc., Chicago, IL, USA) was used for data management and analysis. We used descriptive statistics and binary logistic regression for this work.

### 2.6. Operational Definition

#### 2.6.1. Client Satisfaction

 It is the satisfaction of mothers gained during service delivery. It is the care level gained that increases the likelihood of future utilization maternal health service.

#### 2.6.2. Assessing Level Satisfaction

 A five-point Likert scale ((1) very unsatisfied, (2) unsatisfied, (3) neutral, (4) satisfied, and (5) very satisfied) was used. For the overall satisfaction level, those who will be satisfied in greater than or equal to 75% of the items were categorized as satisfied and those who were satisfied in less than 75% of the items were categorized as unsatisfied [11].

#### 2.6.3. Women Friendly Care

Approach focuses on the rights of women to have access to quality care for themselves as individuals and as mothers and for their infantsand all health providers consider respectful care and support women's emotional, psychological, and social well-being during child birth [13].

### 2.7. Ethical Issues

Before the study was conducted, ethical clearance was obtained from Institutional Research Ethical Review Committee of Arba Minch University. After getting ethical clearance, written permission was obtained from Zonal Health Department and from the woreda health offices. Informed verbal consent from each study participant was obtained after explaining the purpose of the study. Individuals were given the right to participate on voluntary basis and if they did not volunteer to continue from the beginning or at any stage of the interview, they were given the right to withdraw from the study without any consequences. Privacy and strict confidentiality were maintained during the interview process. The information that was collected from this research was kept confidential and stored in files, which did not have participant name on it, but a code number assigned to it, and it was not revealed to anyone except the principal investigator.

## 3. Results

### 3.1. Sociodemographic Result

A total of four hundred thirty (430) postnatal mothers participated in this study, 181 (42.1%) from three hospitals and 249 (57.9%) from ten health centers. More than half of the respondents (246) (57.2%), were Gamo by ethnicity, 262 (60.9%) were Protestant by religion, and 220 (51.2%) were between 25 and 34 years. The mean age was 25.1 years (SD, 5.0). Almost all 414 (96.3%) were married, and half (219) (50.9%) lived in urban areas. The average monthly income was 898 ETB (Table 1).

### 3.2. Obstetric Characteristics of Respondents

For 385 (91.6%) the age at first pregnancy was above 18 years, 225 (52.5%) were gravida of 2–5, and about half of the respondents (49.5%) were para of 2–5. For most of the mothers, 238 (55.3%), the labor lasted up to 12 hours. 59.3% of the mothers did not have previous health facility delivery experience, but 402 (93.5%) of them had visited health facilities for ANC for the recent pregnancy, and 333 (77.4%) came to the health institution without referral for the current delivery (Table 2). 73 (17%) had an unwanted birth, 30 (7%) did have at least a single episode of previous newborn death, 69 (16%) had faced previous abortion, and 28.6% did not possess TV or radio.

### 3.3. Health Facility Related Characteristics

**Figure 1:**
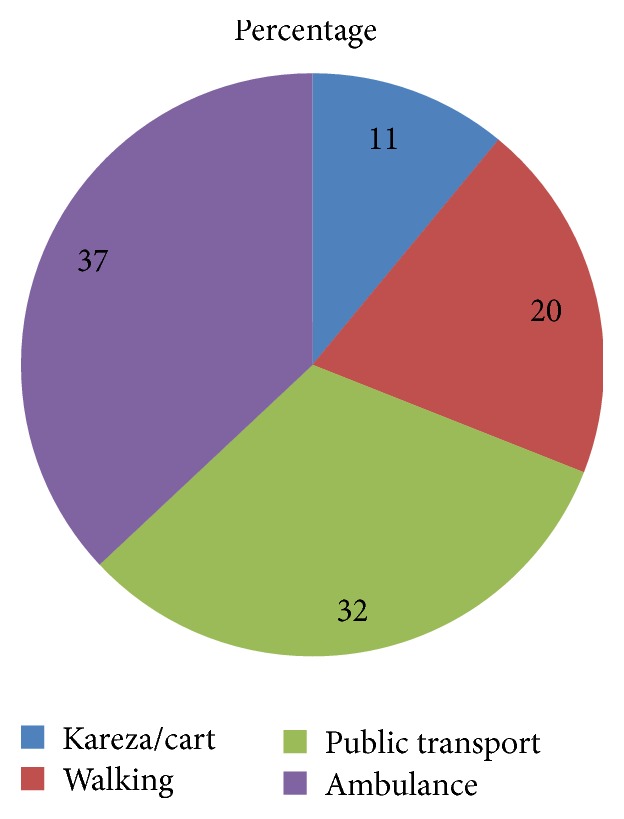
Mode of transportation used by clients to reach facilities where they received delivery care service in public facilities of Gamo Gofa Zone, December 17, 2013, to January 27, 2014.

About three hundred sixty (82.9%) participants travelled for up to one hour, 31 (7.1%) travelled for one to two hours, and 41 (10.1%) travelled for more than two hours to access health institutions to give birth. 158 (36.7%) used ambulance (Figure 1). Most of the patients, 407 (94.3%), waited for less than thirty minutes until being observed or examined by health professional. 232 (54%) patients did not pay for any of the services they received, while 198 (45%) paid for drugs, supplies, and laboratory investigations at the hospitals.

399 (92.8%) of mothers were willing to recommend the facility to a family or a friend. Most of the respondents (355) (82.6%) said there was no shower service, but all of the clients (430) (100%) said toilet has been available in the health institutions.

### 3.4. Client Satisfaction on Delivery Care Service

**Figure 2:**
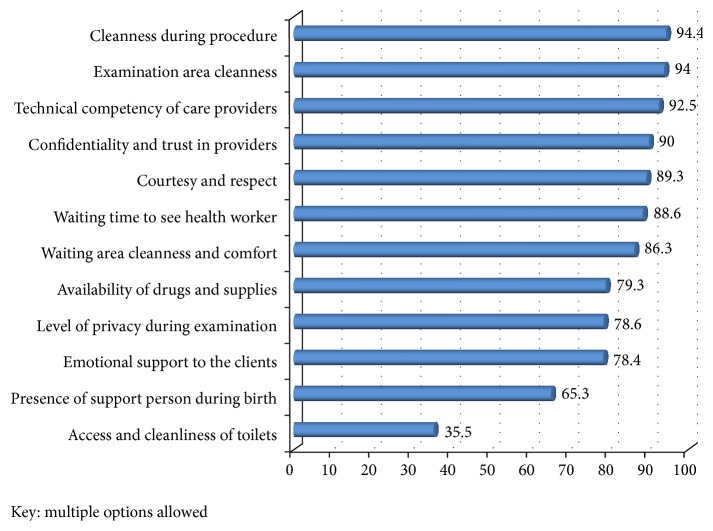
Proportion of client's satisfaction levels with major dimensions of care in 13 public health facilities of Gamo Gofa Zone.

The proportion of mothers who were satisfied with delivery care was 340 (79.1%), varying from 105 (58%) in hospitals and 235 (94.4%) in health centers (*χ* ^2^ = 83.7, df = 12, *P* < 0.001). The proportion of women who complained about an unfriendly attitude or unrespectful care from health workers was higher in the hospitals (*χ* ^2^ = 27.4, df = 1, *P* < 0.001).  Figure 2 shows that the satisfaction levels for cleanliness of toilet (151) (35.1%), presence of relatives or family to support a woman during child birth 281(65.3%), and emotional support to the client during child birth 337(78.4%) were the first three major factors that make mothers satisfied.

### 3.5. Factors Associated with Institutional Delivery Service Satisfaction

In the bivariate analysis educational, occupational, and also institutional related factors were significantly associated with overall satisfaction (Table 3). In order to control the effect of confounders a multiple logistic regression was done. The factors included in the model were those that showed association at the binary logistic regression analysis at a cut-off value (*P* value ≤ 0.25). Presence of support person during child birth was significantly associated with institutional delivery service satisfaction; those who are accompanied by supportive person during birth were 6 times more satisfied than their counterparts (AOR = 6.23 95% CI; 2.75–14.1) and participants who deliver with caesarean section were four times more likely satisfied than those who deliver vaginally (AOR 3.6 95% CI; 1.44–9.06). On the contrary paying participants 73% were less likely satisfied than nonpaying participants (AOR = 0.27 95% CI; 0.09–0.81).

## 4. Discussion

The present study determined the level of client satisfaction at health delivery institutions in Gamo Gofa Zone, southern Ethiopia. The overall satisfaction level was 79.1%. It is lower than a study conducted in west India (86%) [14], but it is comparable to a study conducted in Wolaita (82.9%) in Ethiopia [11] and higher than a study conducted in northern part of Ethiopia (61.9%) [10]. This variation may be due to study setting difference; different research revealed that patient satisfaction is significantly decreased in hospitals or this variation may be because of a real difference in quality of services provided and also could be attributed to study period difference [10, 15].

The result of this study also showed that the proportion of the women attending health centers was more satisfied compared to women attending hospitals (94% and 58%), respectively. The reason for this more satisfaction might be due to the proximity of the health facility. But this finding did not show the association with satisfaction. It could be due to the vast majority of respondents being satisfied with health institution delivery. This study revealed 10.9% women in the study area complained about courtesy and respect from health workers during the course of labor. This was 20.45% in hospitals compared to health centers, only 4.01%. This prevalence was comparable to study done in Addis Ababa that was 12.4% (14.8,9.4 %) in hospitals and health centres, respectively [16].

In the current study satisfaction of delivering mothers was predicted by presence of support person during child birth, mode of delivery, and payment status. This was consistent with other studies [17–19]. However the level of satisfaction was not affected by either age, income, or educational status; this result is in contrast to a study performed in other settings in Ethiopia and elsewhere; this may indicate that satisfaction might be more affected by other factors than the sociodemographic characteristics of participants [10, 11, 20].

An important finding from this study is that there was a significant association between presence of support person during child birth and client satisfaction, as reported by others [17, 18, 21]. The possible explanation could be that women need companionship at birth and this may help to reduce fears and anxiety which contribute to the reduction of pain during labor and delivery [22].

Also statically significant association was found between the client satisfaction and mode of delivery. Clients delivering with caesarean section are four times more likely to be satisfied than the other modes of deliveries. Other studies done in 1992 by Stadlmayr and colleagues reported no significant association between modes of delivery and satisfaction. The differences between these studies and our results may suggest differences between populations studied or change in maternal perception for caesarean section after they have been saved from complications [23]. However client satisfaction was reduced if the women had to pay for the services. This is in line with a study conducted in Jimma and other studies in Ethiopia [19, 24]. This variation may be because their expectation of the delivery service payment may be free in all health institutions but they paid in hospital indirectly for drugs and laboratory investigations and also socioeconomic status difference in the study population may be the cause for their dissatisfaction.

## 5. Limitations of the Study

Social desirability bias might have affected the quality of data collected because study subjects might face difficulty in responding to dissatisfaction in the presence of data collectors. But data collection was done in a private room by nonstaff midwives to reduce the bias. The other limitation comes from the institution based nature of the study which makes inferring to all delivering mothers in the study area since most of the deliveries take place at night indicating a need for further study by using a more representative sample and also the nature of the study design temporal relations cannot be drawn. Besides the above shortcomings, the study generated important data that can be used as an input for improvement of maternal health services and with increased satisfaction in the study area.

## 6. Conclusions

Nowadays client satisfaction plays a significant role in increasing utilization of women for institution based delivery and also it is necessary to improve quality of health care in reducing maternal morbidity and mortality. The study shows that overall satisfaction level is good, but there is room for improvements; more emphasis should be on giving women friendly care, particularly at hospitals.

## Figures and Tables

**Table 1:** Sociodemographic characteristic of respondents in public health facility of Gamo Gofa Zone, southwest Ethiopia, May 2014.

Variable	Frequency (*n* = 430)	Percent (%)
Age		
15–24	186	43.3
25–34	220	51.2
34–44	24	5.6
Residence		
Urban	219	50.9
Rural	211	49.1
Religion		
Protestant	262	60.9
Orthodox	148	34.4
Muslim	20	4.7
Education		
Illiterate	121	28.1
Primary	124	28.8
Secondary	122	43.1
Above secondary	63	13.7
Occupation		
Housewife	262	60.9
Government employed	45	10.5
Merchant	44	10.2
Daily labourer	42	9.8
Other	37	8.6
Monthly income		
<500 ETB	70	16.8
500–1500 ETB	252	58.6
>1500 ETB	108	25.5
Ethnicity		
Gamo	246	57.2
Gofa	75	17.4
Other	64	14.3
Delivery took place		
Hospitals	181	42.1
Health centers	249	57.9

**Table 2:** Obstetric characteristics of respondents in Gamo Gofa Zone, southwest Ethiopia, May 2014.

Variables	Frequency (*n* = 430)	Percent (%)
Age at first marriage		
<18	116	27
>18	314	73
Age at first pregnancy		
>18	74	17.2
<18	356	82.8
Gravidity		
One	160	37.2
Two–five	225	52.3
>five	45	10.5
Parity		
One	181	42.1
Two–five	213	49.5
>five	36	8.4
Reason for visit		
Planned	333	77.4
Referred	97	22.6
Status of pregnancy		
Wanted	345	81.2
Unwanted	81	18.8
Mode of delivery		
SVD	355	82.6
Assisted delivery	36	8.4
CS delivery	39	9.1
Maternal condition after delivery		
Normal	365	84.9
With complication	65	15.1
Foetal outcome		
Live birth	403	93.7
Stillbirth	27	6.3
Ever had neonatal death		
Yes	30	7
No	400	93
Ever had still birth		
Yes	34	93
No	396	79
Duration of last delivery (hr)		
<12	238	55.5
12–24	143	33.3
>24	49	11.4

**Table 3:** Predictors of satisfaction in public health facility of Gamo Gofa Zone, southwest Ethiopia, May 2014 (*n* = 430).

Variable	Satisfied	Unsatisfied	COR (95% CI)	AOR (95% CI)
Education				
Illiterate	86 (20.0%)	35 (8.6%)	1	1
Primary	106 (24.7%)	18 (4.2%)	2.4 (1.27–4.52)^*∗*^	1.56 (0.68–3.5)
Secondary	101 (23.5%)	21 (4.9%)	2.4 (1.27–4.52)^*∗*^	1.65 (0.71–3.87)
Above secondary	47 (10.9%)	16 (3.7%)	1.12 (0.6–2.34)	1.01 (0.23–3.40)
Occupation				
Housewife	211 (49.1%)	46 (10.7%)	1	1
Government employed	34 (7.9%)	11 (2.6%)	0.68 (0.32–1.43)	1.33 (0.49–3.55)
Self-employed	61 (14.2%)	24 (5.6%)	0.56 (0.32–0.98)^*∗*^	0.85 (0.24–2.99)
Other	34 (7.9%)	9 (2.1%)	0.83 (0.37–1.84)	0.77 (0.25–2.29
Mode of delivery				
SVD	286 (67.2%)	66 (15.3)	1	1
Assisted delivery	19 (4.4%)	17 (4.0%)	0.26 (0.12–0.52)^*∗*^	0.59 (0.26–1.34)
CS	32 (7.4%)	7 (1.6%)	1.05 (0.45–2.47)	3.61 (1.44–9.06)^*∗*^
ANC follow-up				
Yes	323 (75.1%)	79 (18.4%)	1	1
No	17 (4%)	11 (2.6%)	0.37 (0.17–0.83)^*∗*^	0.52 (0.21–1.36)
Payment status				
Paid	119 (27.7%)	11 (2.6%)	0.07 (0.038–0.15)^*∗*^	0.27 (0.09–0.81)^*∗*^
Free	221 (51.4%)	79 (18.4%)	1	1
Distance				
<half an hour	189 (44.0%)	40 (9.3%)	1	1
>half-one hour	109 (32.1%)	24 (5.6%)	0.36 (0.18–0.71)^*∗*^	1.81 (0.72–4.56)
1 hour-two hours	13 (3.0%)	9 (2.1%)	0.8 (0.18–0.79)^*∗*^	1.98 (0.75–5.16)
>two hours	29 (6.7%)	17 (4.0%)	19 (0.42–3.34)	0.73 (0.19–2.72)
Presence of support person during birth				
Yes	191 (44.4%)	8 (1.9%)	13.14 (6.17–28)^*∗*^	6.23 (2.75–14.11)^*∗*^
No	149 (34.7%)	82 (19.1%)	1	1
Delivery took place				
Hospitals	105 (58%)	76 (42%)	1	1
Health centers	235 (94.4%)	14 (5.6%)	12.15 (6.57–22.4)^*∗*^	2.76 (0.99–7.6)

^*∗*^Statistically significant associations.
